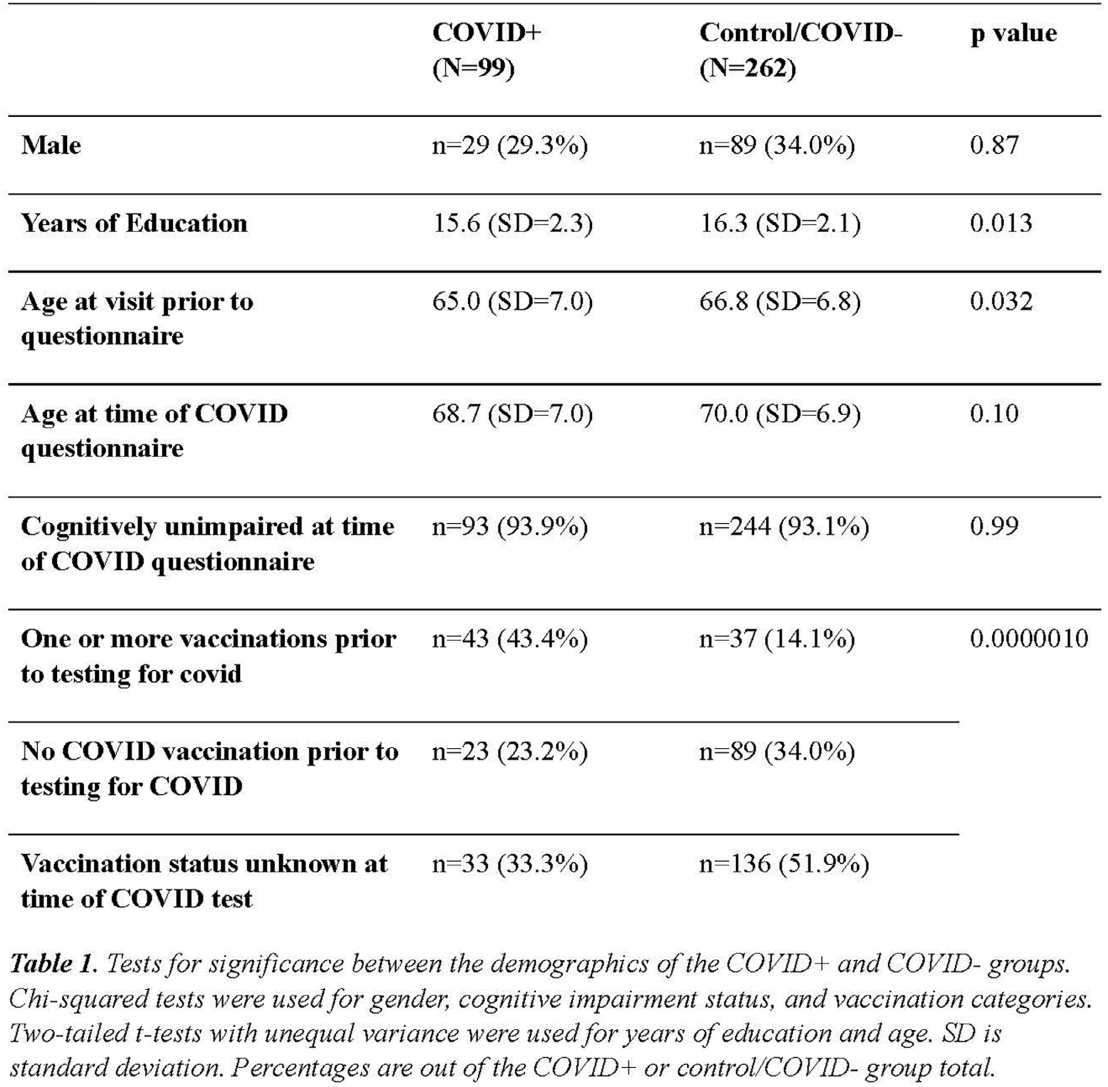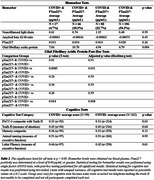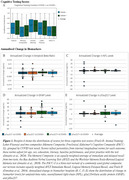# Association of COVID‐19 Positivity with Biomarkers of Alzheimer's Disease and Cognitive Test Performance

**DOI:** 10.1002/alz70856_102887

**Published:** 2025-12-25

**Authors:** Sarah A Scalzo, Elias G Seath, Rachel L Studer, Erin M. Jonaitis, Elizabeth M. Planalp, Ramiro Eduardo Rea Reyes, Rachael E. Wilson, Sterling C. Johnson, Rebecca E. Langhough

**Affiliations:** ^1^ Department of Medicine, Division of Geriatrics and Gerontology, University of Wisconsin School of Medicine and Public Health, Madison, WI, USA; ^2^ Wisconsin Alzheimer's Institute, University of Wisconsin‐Madison School of Medicine and Public Health, Madison, WI, USA; ^3^ Wisconsin Alzheimer's Institute, University of Wisconsin School of Medicine and Public Health, Madison, WI, USA; ^4^ Waisman Center, University of Wisconsin‐Madison, Madison, WI, USA; ^5^ Wisconsin Alzheimer's Disease Research Center, Madison, WI, USA; ^6^ Department of Medicine, University of Wisconsin‐Madison School of Medicine and Public Health, Madison, WI, USA; ^7^ Wisconsin Alzheimer's Disease Research Center, University of Wisconsin School of Medicine and Public Health, Madison, WI, USA; ^8^ Wisconsin Alzheimer's Institute, University of Wisconsin School of Medicine and Public Health, Madison, WI, USA

## Abstract

**Background:**

The amyloid cascade that causes Alzheimer's disease (AD) is associated in part with neuroinflammation, which may be affected by peripheral disease. COVID‐19 has been implicated in cognitive change, though its effect on AD biomarkers is unclear. We leverage a cohort with biomarker and cognitive data pre‐ and post‐COVID‐pandemic to examine these relationships.

**Method:**

Data was obtained from the Wisconsin Registry for Alzheimer's Prevention (Table 1). Participants return biennially for cognitive and biomarker testing. We used the data from the most recent pre‐pandemic visit, compared with the first post‐pandemic visit, to examine biomarker and cognitive change. One‐way ANOVA was used to examine annualized biomarker change as a function of both pTau217 positivity and participant‐reported prior COVID test‐positivity (pTau217‐/COVID‐; pTau217‐/COVID+; pTau217+/COVID‐; pTau217+/COVID+). Baseline‐adjusted cognitive scores on tests measuring cognitive processes previously reported to be affected by COVID (trails B, animal naming, and letter fluency tests, memory and PACC‐3 composite scores) were compared using two‐tailed, unpaired t‐tests (COVID‐ vs COVID+).

**Result:**

The pTau217+/COVID‐ group differed from both the pTau217‐/COVID‐ and pTau217‐/COVID+ groups, but these differences did not survive correction for multiple comparisons (Table 2). The biomarkers did not show any other significant differences between COVID+ and COVID‐ groups. The cognitive testing results trended towards the opposite relationship found in past studies, with those who were COVID+ averaging non‐significantly higher percentile scores (Figure 1). Participants who tested positive for COVID‐19 were more likely to be vaccinated, likely due to the increase in positive tests after vaccines were available.

**Conclusion:**

Overall, we did not find significant associations between COVID‐19, cognitive test scores, or biomarker levels. Our data may be less robust given the high percentage of vaccinations among COVID‐19 positive cases and that vaccination decreases infection severity. It is also possible that the slightly younger age of the COVID+ group acted as a confounding variable. Strengths of our study include comparison of individual biomarker data before and after the spread of COVID‐19, interpretation of individual cognitive test scores relative to previous performance, and a large cohort size enriched with Alzheimer's disease risk factors.